# Immunogenicity of four doses of oral poliovirus vaccine when co-administered with the human neonatal rotavirus vaccine (RV3-BB)

**DOI:** 10.1016/j.vaccine.2019.09.071

**Published:** 2019-11-20

**Authors:** Daniel Cowley, Rini Mulia Sari, Amanda Handley, Emma Watts, Novilia S. Bachtiar, Jarir At Thobari, Cahya Dewi Satria, Nada Bogdanovic-Sakran, Hera Nirwati, Francesca Orsini, Katherine J. Lee, Carl D. Kirkwood, Yati Soenarto, Julie E. Bines

**Affiliations:** a Enteric Diseases, Murdoch Children’s Research Institute, Parkville, Victoria, Australia; b Department of Paediatrics, The University of Melbourne, Parkville, Victoria, Australia; c Bio Farma PT, Bandung, Indonesia; d Medicines Development for Global Health, Melbourne, Victoria, Australia; e Departments of Pharmacology and Therapy, Public Health and Nursing, Universitas Gadjah Mada, Yogyakarta, Indonesia; f Departments of Microbiology, Public Health and Nursing, Universitas Gadjah Mada, Yogyakarta, Indonesia; g Paediatric Research Office, Department of Paediatrics Faculty of Medicine, Public Health and Nursing, Universitas Gadjah Mada, Yogyakarta, Indonesia; h Clinical Epidemiology and Biostatistics Unit and the Melbourne Children’s Trials Centre, Murdoch Children’s Research Institute, Parkville, Victoria, Australia; i Bill and Melinda Gates Foundation, Seattle, WA, USA; j Department of Gastroenterology and Clinical Nutrition, Royal Children’s Hospital, Parkville, Victoria, Australia

**Keywords:** Rotavirus, Poliovirus, Neonatal, Vaccine

## Abstract

**Background:**

The RV3-BB human neonatal rotavirus vaccine was developed to provide protection from severe rotavirus disease from birth. The aim of this study was to investigate the potential for mutual interference in the immunogenicity of oral polio vaccine (OPV) and RV3-BB.

**Methods:**

A randomized, placebo-controlled trial involving 1649 participants was conducted from January 2013 to July 2016 in Central Java and Yogyakarta, Indonesia. Participants received three doses of oral RV3-BB, with the first dose given at 0–5 days (neonatal schedule) or ~8 weeks (infant schedule), or placebo. Two sub-studies assessed the immunogenicity of RV3-BB when co-administered with either trivalent OPV (OPV group, n = 282) or inactivated polio vaccine (IPV group, n = 333). Serum samples were tested for antibodies to poliovirus strains 1, 2 and 3 by neutralization assays following doses 1 and 4 of OPV.

**Results:**

Sero-protective rates to poliovirus type 1, 2 or 3 were similar (range 0.96–1.00) after four doses of OPV co-administered with RV3-BB compared with placebo. Serum IgA responses to RV3-BB were similar when co-administered with either OPV or IPV (difference in proportions OPV vs IPV: sIgA responses; neonatal schedule 0.01, 95% CI −0.12 to 0.14; p = 0.847; infant schedule −0.10, 95% CI −0.21 to −0.001; p = 0.046: sIgA GMT ratio: neonatal schedule 1.23, 95% CI 0.71–2.14, p = 0.463 or infant schedule 1.20, 95% CI 0.74–1.96, p = 0.448).

**Conclusions:**

The co-administration of OPV with RV3-BB rotavirus vaccine in a birth dose strategy did not reduce the immunogenicity of either vaccine. These findings support the use of a neonatal RV3-BB vaccine where either OPV or IPV is used in the routine vaccination schedule.

## Highlights

• Both OPV and rotavirus vaccines contain live, attenuated strains that replicate in the gut.
• OPV and rotavirus vaccine co-administration has been associated with lower rotavirus immune responses.
• RV3-BB is a novel human neonatal rotavirus vaccine that provides protection from rotavirus disease from birth.
• OPV and RV3-BB co-administration did not reduce immunogenicity of either vaccine.
• These findings support the use of RV3-BB where either OPV or IPV is used in the routine schedule.

## 1 Introduction

The rotavirus vaccines Rotarix (GlaxoSmithKline Biologicals, London, UK) and RotaTeq (Merck & Co, Kenilworth, NJ, USA) have significantly reduced child mortality from gastroenteritis [13], [3]. Despite this, over 90 million infants still lack access to a rotavirus vaccine [11]. The recent WHO prequalification of Rotavac (Bharat Biotech, Hyderabad, India) and Rotasiil (Serum Institute of India, Pune, India) may alleviate cost and supply barriers, however there remains the challenge of sub-optimal efficacy of the current vaccines in low-income countries [9].The human neonatal rotavirus vaccine, RV3-BB, is in clinical development with a birth dose vaccination schedule to address some of these challenges. A randomized, placebo-controlled trial undertaken in Central Java and Yogyakarta, Indonesia demonstrated the efficacy of RV3-BB when given in a neonatal (first dose 0–5 days of age) or infant (first dose 8–10 weeks of age) schedule [2].

The implementation of RV3-BB with a birth dose requires co-administration with other vaccines in the Expanded Program on Immunization (EPI) schedule. The oral polio vaccine (OPV) is administered at birth in many developing countries. The global effort to eradicate poliovirus has predominately used the trivalent OPV (tOPV), which includes attenuated strains of types 1, 2 and 3. Following the global eradication of type 2 poliovirus, a switch to bivalent OPV (bOPV), containing poliovirus 1 and 3, and monovalent OPV (mOPV), containing type 1 only, has occurred. A complete switch from OPV to the inactivated polio vaccine (IPV) is planned [21], [20]. However, the continued circulation of wild-type poliovirus type 1, and programmatic difficulties necessitate that both bOPV and mOPV remain administered in many developing countries [10]. The successful co-administration of RV3-BB and OPV would facilitate the introduction of RV3-BB into national immunization programs.

Both OPV and live oral rotavirus vaccines have a common route of administration and both replicate in the gut, therefore the potential for interference exists. The co-administration of tOPV with Rotarix and RotaTeq is associated with lower immunogenicity and stool shedding particularly at the first rotavirus vaccine dose compared with staggered administration (vaccines administered more than a day apart) [6], [15], [12]. Similarly, the co-administration of Rotarix with bOPV and mOPV is associated with lower rotavirus immunogenicity [8]. There appears to be no interference on sero-protective rates to poliovirus types 1, 2 and 3 antibody following co-administration of OPV and Rotarix and RotaTeq vaccines [6], [15]. However, it is unknown if co-administration of RV3-BB and OPV in a birth dose schedule will affect the immunogenicity of either vaccine. During the Phase IIb efficacy trial of RV3-BB, two immunogenicity sub-studies were conducted with participants co-administered OPV (n = 333) or IPV (n = 282) in the routine vaccine schedule. The aim of the present study is to compare the sero-protective response to poliovirus types 1, 2 and 3 following the first and fourth dose of OPV when co-administered with the two RV3-BB vaccination schedules between the vaccine and placebo groups (Sub-study B). Secondly, to describe serum anti-rotavirus immunoglobulin A (IgA) response following doses 1 and 4 of RV3-BB when RV3-BB was given in a neonatal or infant schedule and co-administered with either IPV or OPV, compared with placebo (Sub-study A and B).

## 2 Materials and methods

### 2.1 Trial design and recruitment

The study design, recruitment, randomisation and follow-up of the Phase IIb efficacy, safety and immunogenicity of the RV3-BB vaccine has been previously described [2]. Briefly, the study was a randomized, double-blind, placebo-controlled trial involving 1649 participants conducted from January 2013 to July 2016 in primary health centres and hospitals in Central Java and Yogyakarta, Indonesia. The protocol was approved by the ethics committees of Universitas Gadjah Mada, Royal Children's Hospital Melbourne and National Agency of Drug and Food Control, Republic of Indonesia (Australian New Zealand Clinical Trials Registry number: ACTRN12612001282875). Eligible infants (healthy, full term babies 0–5 days of age, birth weight of 2.5–4.0 kg) were randomized into one of three groups (neonatal vaccine group, infant vaccine group, or placebo group) in a 1:1:1 ratio according to a computer generated code (block size = 6) stratified by province.

### 2.2 Vaccines

**Fig. 1:**
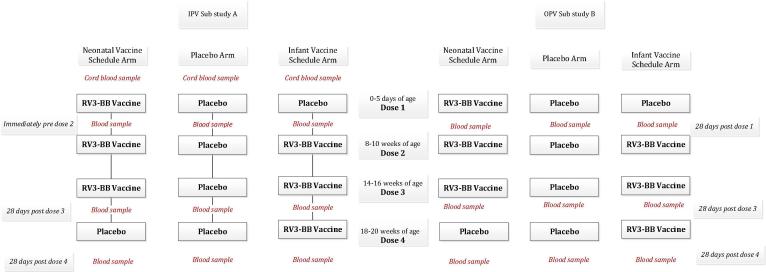
Study Design with blood collection time-points for Sub-study A and Sub-study B.

The investigational product (IP) consisted of oral RV3-BB vaccine or placebo. RV3-BB clinical trial lots were prepared at Meridian Life Sciences (Memphis, USA) to a titre of 8.3–8.7 × 10^6^ FFU/mL in serum free media supplemented with 10% w/v sucrose. Participants received four 1 ml oral doses of IP (vaccine or placebo) according to their treatment allocation, with doses administered at 0–5 days (IP dose 1), 8–10 weeks (IP dose 2), 14–16 weeks (IP dose 3) and 18–20 weeks of age (IP dose 4) (Fig. 1). In the neonatal vaccine group IP doses 1, 2, and 3 were RV3-BB and dose 4 was placebo, and in the infant vaccine group, IP dose 1 was placebo and doses 2, 3, and 4 were RV3-BB. In the placebo group all four IP doses consisted of media with 10% w/v sucrose and was visually indistinguishable from the vaccine. IP doses 2, 3 and 4 (vaccine or placebo) were preceded by a 2 ml dose of antacid solution (Mylanta® Original). Feeding was withheld for 30 min before and after each dose.

### 2.3 Two sub-studies were recruited during the trial

**Fig. 2:**
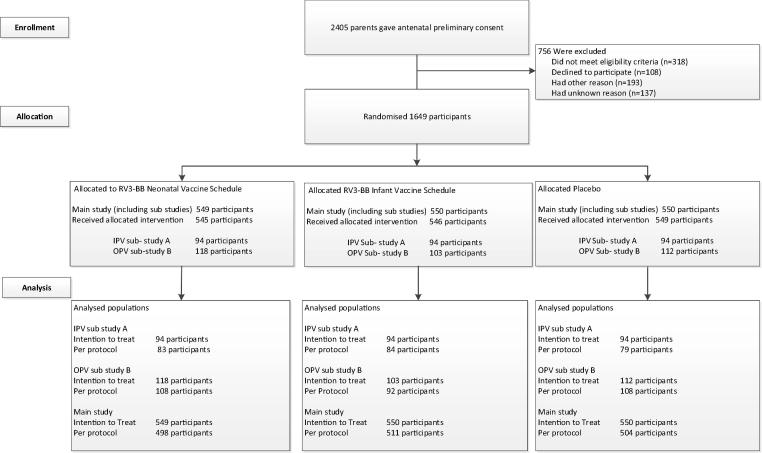
Consort diagram of participant randomization, trial assignment and follow-up.

Sub-study A (IPV group): The primary objective of sub study A was to describe the sIgA response following 3 doses of RV3-BB when co-administered with IPV. Sub-Study A (IPV group) consisted of 282 participants who were administered IPV (Imovax Polio, Sanofi Pasteur, Lyon, France) at 8, 12, 16 and 36 weeks of age as described by the Indonesian EPI schedule (Fig. 1, Fig. 2). The first cohort of participants (n = 282) recruited into the main study were enrolled into Sub-study A. Enrolment into Sub-study A occurred in clinical sites where IPV was administered in the routine EPI program as part of a regional IPV Demonstration Project.

Sub-Study B: (OPV group): The primary objective for Sub-study B was to describe the proportion of infants with a positive sero-protective response against polio strains 1–3 after four doses of OPV when co-administered with RV3-BB (Fig. 1, Fig. 2). A secondary objective was to measure the serum anti-rotavirus IgA sero-response following 3 doses of RV3-BB when co-administered with OPV. Sub-study B consisted of 333 participants who were co-administered 4 doses of tOPV (Sabin trivalent OPV, PT Bio Farma, Bandung Indonesia) with IP doses 1, 2, 3 and 4 within 24 h. Participants in both sub-studies received other vaccines according to the Indonesian EPI schedule.

### 2.4 Serum collection and RV3-BB vaccine immunogenicity

Serum anti-rotavirus IgA antibody titres were measured using previously described methods [7], [1]. The difference in the timing of the blood collection for the IPV and OPV groups (Fig. 1) reflected the differences in primary objective for each of the two sub-studies.

In the IPV group, serum anti-rotavirus IgA immune response was assessed in blood collected from the cord (at birth), immediately prior to IP dose 2 (8–10 weeks), 28 days post IP dose 3 (18–20 weeks) and 28 days post IP dose 4 (22–24 weeks) (Fig. 1). The baseline for sero-response in the neonatal schedule was the sIgA level in cord blood for each participant. Baseline for the infant schedule was blood collected immediately prior to IP dose 2.

In OPV group, blood was collected 28 days post IP dose 1 (~4 weeks), 28 days post dose IP dose 3 (18–20 weeks) and 28 days post IP dose 4 (22–24 weeks) (Fig. 1). Polio seropositivity following a birth dose of OPV is generally assessed 28 days following the dose so blood collection was conducted at that time-point. For the assessment of sIgA in the neonatal schedule co-administered with OPV the baseline values were assumed to be below the lower limit of detection based on previous data [4], [1]. In the infant schedule, baseline values were obtained from analysis of sera collected 28 days post IP dose 1 (~4 weeks), or assumed to be below the lower limit of detection if the sample was missing.

### 2.5 Poliovirus neutralization assay in the OPV group

Serum samples were tested for poliovirus antibodies by neutralization assays conducted at PT Bio Farma (Persero), Bandung, Indonesia using standardized Centers for Disease Control protocol [19]. Each sample was tested for antibodies to poliovirus types 1, 2 and 3 in triplicate and the mean reciprocal neutralizing antibody titre reported. A sero-protective response was defined as a reciprocal neutralizing antibody titre ≥ 8.

### 2.6 Statistical methods

In sub-study A, the sample size was calculated based on the comparison of cumulative vaccine take following three doses of RV3-BB vaccine in each of the neonatal and the infant vaccine schedule groups with the placebo group. Assuming 25% (estimate based on studies in low income settings) of participants in placebo group demonstrate a positive vaccine take, 85 participants would be required per treatment arm to reject the null hypothesis of no difference with either vaccine if 50% of vaccinated participants demonstrate a vaccine take (based on a two-sided test with α = 0.05). Allowing for 10% non-adherence to the per protocol population a total of 282 infants were required (94 in each of the 3 treatment arms).

In sub-study B, the primary objective was to describe the proportion of infants with a positive sero-protective response against polio strains 1–3 post 4 doses of OPV in each of the 3 intervention arms. Previous studies estimated that approximately 80–90% of infants receiving OPV demonstrate a positive sero-protective response to polio strains 1–3, both for those receiving no rotavirus vaccine and in infants receiving one of the licensed rotavirus vaccines. One hundred infants per treatment arm would allow an estimation of the proportion with a positive sero-protective response to within 8% based on 80% with a positive response, and within 6% based on 90% with a positive response (reporting results as a 2-sided 95% confidence interval [CI]). Allowing for 10% non-adherence to the per protocol population, we therefore aim to recruit a total of 333 participants (111 per treatment arm).

The number and proportion of participants with a sero-protective response to poliovirus types 1, 2 and 3 28 days post IP dose 1 (~4 weeks) and 28 days post IP dose 4 (22–24 weeks) are summarised in the OPV group by treatment group and are presented with 95% CI. Serum antibody titres to each poliovirus type at each time point are summarised as the geometric mean and its 95% CI by treatment group. Serum antibody titres to OPV are combined for placebo and infant vaccine groups 28 days post IP dose 1 (~4 weeks) as both groups received placebo at this time point. Outcomes were excluded in participants that did not receive all four doses of OPV and those with missing data.

Serum anti-rotavirus IgA concentrations prior to IP dose 2 (8–10 weeks) and 28 days post IP dose 4 (22–24 weeks) are summarised as the geometric mean by treatment group. Differences in antibody titre between each of the vaccine groups and the placebo group are presented as geometric mean ratios and their 95% CIs. Serum anti-rotavirus IgA response (defined as a ≥3 fold increase in titre from baseline following administration of IP) is summarised as the number and proportion of participants with a serum anti-rotavirus IgA response at each serum collection time point along with its 95% CI in those with available data. Cumulative serum anti-rotavirus IgA response (defined as a response following dose of 1, 2 or 3 of IP for the neonatal vaccine group, and following doses 2, 3 or 4 of IP for the infant vaccine group) are summarised similarly. The different schedules of vaccination used in the neonatal and infant groups resulted in two definitions of the placebo group (defined as dose of 1, 2 or 3 of placebo labelled as the neonatal placebo group, and doses 2, 3 or 4 of placebo labelled as the infant vaccine group). A Chi-Squared test was used to compare the difference in proportions with a positive response to RV3-BB at each time point between treatment groups, with group differences presented as differences in proportions along with their 95% CIs. The statistical analysis of anti-rotavirus IgA was performed on the per-protocol population (having received all doses of IP within the pre-defined visit windows) [2].

## 3 Results

### 3.1 Study population

There were 333 participants recruited into OPV group. The per protocol population (n = 308) consisted of 108 participants who received the neonatal vaccine schedule, 92 the infant vaccine schedule and 108 the placebo. The majority (>95%) of OPV doses were administered within 23 h of the IP (Fig. 2).

There were 282 participants recruited into IPV group. The analysis presented was performed on the per protocol population (n = 246), of whom 83 participants received the neonatal vaccine schedule, 84 the infant vaccine schedule and 79 the placebo (Fig. 2).

The demographic characteristics at baseline and the age of receipt of the first dose of vaccine or placebo were similar across the three randomised groups in both the OPV and IPV groups (Table 1).

**Table 1:** Demographics of participants in the 3 randomised arms in the OPV (oral polio vaccine) and IPV (inactivated polio vaccine) group (per protocol population).

	OPV group (n = 308)			IPV Group (n = 246)		
	Neonatal Vaccine group (n = 108)	Infant Vaccine group (n = 92)	Placebo (n = 108)	Neonatal Vaccine group (n = 83)	Infant Vaccine group (n = 84)	Placebo (n = 79)
Age at randomization in days: Mean (SD)	3.3 (1.4)	3.3 (1.2)	3.2 (1.4)	3.2 (1.2)	3.1 (1.2)	3.2 (1.1)
Sex – n (%) Male	61 (56%)	43 (47%)	62 (57%)	47 (57%)	47 (56%)	37 (47%)
Ethnicity – n (%):						
Javanese	108 (100%)	92 (100%)	107 (99%)	83 (100%)	84 (100%)	79 (100%)
Other	0 (0%)	1 (1%)	0 (0%)	0 (0%)	0 (0%)	
Gestational age in weeks: Mean (SD)	39.47 (1.10)	39.74 (0.98)	39.62 (1.03)	39.66 (1.13)	39.46 (1.03)	39.54 (1.12)
Birth weight in grams: Mean (SD)	3190.74 (370.98)	3203.80 (331.76)	3100.46 (341.55)	3079.52 (329.73)	3126.55 (324.29)	3101.90 (325.95)
Height/Length in cms: Mean (SD)	48.54 (1.49)	48.62 (1.30)	48.39 (1.68)	48.68 (1.74)	48.30 (1.98)	48.74 (1.76)

### 3.2 Response to poliovirus vaccination in the OPV group

The proportion of participants with a sero-protective response to poliovirus types 1, 2 and 3 following doses 1 and 4 of OPV were similar in the neonatal and infant vaccine groups and when compared to placebo (Table 2). Sero-protective responses to each poliovirus type were 96–100% post 4 doses of IP irrespective of the vaccination schedule of RV3-BB or whether the participants received placebo. The GMTs of antibodies to each poliovirus type following 1 and 4 doses OPV were similar for each of the RV3-BB vaccine schedules with overlapping 95% confidence intervals, and were similar to the placebo group (Table 3).

**Table 2:** Seroprotective responses to poliovirus type 1, 2 and 3 following co-administration of oral polio vaccine (OPV) and RV3-BB vaccine.

Polio strain and OPV Dose number	Neonatal Vaccine Schedule	Infant Vaccine Schedule	Placebo*
**Polio type 1**			
Post dose 1 of OPV	96/105 (0.91, 0.84–0.96)	N/A	185/198 (0.93, 0.89–0.96)
Post dose 4 of OPV	105/105 (1.00, 0.97–1.00)	85/88 (0.96, 0.90–0.99)	103/103 (1.00, 0.96–1.00)
**Polio type 2**			
Post dose 1 of OPV	103/105 (0.98, 0.93–1.00)	N/A	191/198 (0.96, 0.93–0.99)
Post dose 4 of OPV	105/105 (1.00, 0.97–1.00)	88/88 (1.00, 0.96–1.00)	103/103 (1.00, 0.96–1.00)
**Polio type 3**			
Post dose 1 of OPV	79/105 (0.75, 0.66–0.83)	N/A	139/198 (0.70, 0.63–0.76)
Post dose 4 of OPV	105/105 (1.00, 0.97–1.00)	88/88 (1.00, 0.96–1.00)	101/103 (0.98, 0.93–1.00)

Results presented as the number with a positive response over the number assessed at each time point. Proportion of participants with anti-Poliovirus seroprotective response and 95% confidence interval shown in parenthesis.

* Placebo and Infant schedules are combined for Post dose 1 of OPV as both groups received placebo at this time point. N/A, not available. Participants who did not receive all doses of OPV were excluded from the analysis.

**Table 3:** Antibody titres to poliovirus type 1, 2 and 3 following co-administration of oral polio vaccine (OPV) and RV3-BB vaccine.

Polio strain and OPV Dose number	Neonatal Vaccine Schedule		Infant Vaccine Schedule		Placebo*	
	*N*	GMT	*N*	GMT	*N*	GMT
**Polio type 1**						
Post dose 1 of OPV	105	83.1 (62.9, 109.8)	N/A	N/A	198	77.9 (63.5, 95.5)
Post dose 4 of OPV	105	471.4 (377.8, 588.3)	88	368.8 (276.8, 491.2)	103	495.6 (398.2, 616.8)
**Polio type 2**						
Post dose 1 of OPV	105	155.9 (127.8, 190.1)	N/A	N/A	198	158.8 (135.2, 186.6)
Post dose 4 of OPV	105	720.9 (608.4, 854.2)	88	729.8 (620.5, 858.3)	103	788.5 (668.7, 929.7)
**Polio type 3**						
Post dose 1 of OPV	105	39.0 (28.3, 53.6)	N/A	N/A	198	25.4 (20.9, 30.9)
Post dose 4 of OPV	105	275.6 (224.8, 337.8)	88	249.4 (198.1, 313.9)	103	236.4 (188.1, 297.2)

Results presented are Geometric mean titres (GMT) of antibodies to each poliovirus serotype following dose 1 and dose 4 of OPV, 95% confidence intervals are shown in parenthesis.

* Placebo and Infant schedules are combined for Post dose 1 of OPV as both groups received a placebo at this time point. N/A, not available. Participants who did not receive all doses of OPV were excluded from analysis.

### 3.3 Serum immune response to RV3-BB

**Fig. 3:**
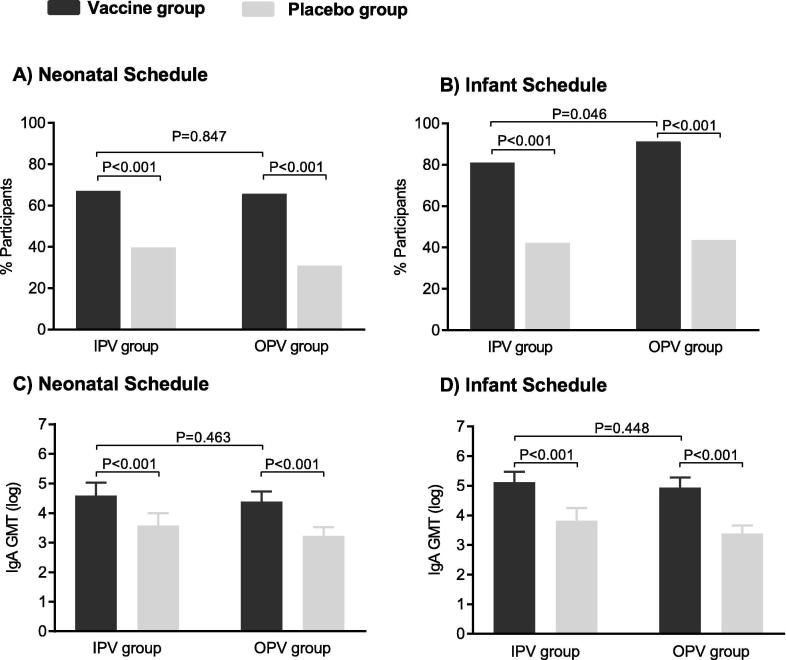
Proportion of participants with a cumulative serum anti-rotavirus IgA response (A and B) and geometric mean titre of serum IgA presented on the natural log scale (C and D) following administration of three doses of RV3-BB in participants receiving IPV and OPV. Error bars represent 95% confidence intervals.

When three doses of RV3-BB were co-administered with OPV in the neonatal vaccine schedule, a serum anti-rotavirus IgA response was detected in 71/108 (66%) participants, compared with 33/107 (31%) in the neonatal placebo group (difference in proportions 0.35, 95% CI 0.22–0.47; p < 0.001) (Fig. 3A). Following three doses of RV3-BB co-administered with OPV in the infant vaccine schedule, serum anti-rotavirus IgA responses were detected in 84/92 (91%) participants compared with 47/108 (43%) in the infant placebo group (difference in proportions 0.48, 95% CI 0.37–0.59; p < 0.001) (Fig. 3B). Similarly, when RV3-BB was administered with IPV, there was a difference in the proportion with a serum anti-rotavirus IgA responses when compared to the placebo group in both the neonatal vaccine (55/82, 67% vs 31/78, 40%; difference in proportions 0.27, 95% CI 0.12–0.42; p < 0.001) and infant vaccine groups (68/84, 81% vs 33/78, 42%; difference in proportions 0.39, 95% CI 0.25–0.52; p < 0.001) (Fig. 3A, B).

Importantly, there was little evidence of a difference in the proportion of participants with a serum anti-rotavirus IgA response to RV3-BB when IPV compared with OPV was used as the poliovirus vaccine when RV3-BB was given in the neonatal vaccine schedule (55/82, 67% vs 71/108, 66%; difference in proportions 0.01, 95% CI −0.12 to 0.14; p = 0.847) (Fig. 3A). In those who received the infant vaccine schedule, the proportion of participants with a serum anti-rotavirus IgA response was lower in those who received IPV compared to those who received OPV, although the difference was small (68/84, 81% vs 84/92, 91%; difference in proportions −0.10, 95% CI −0.21 to −0.001; p = 0.046) (Fig. 3B).

The proportion of participants with a serum anti-rotavirus IgA response following a single dose of RV3-BB in the neonatal vaccine group was low in both immunogenicity sub-studies, with little evidence of a difference between the IPV and OPV groups (17/82, 21% vs 15/107; 14% difference in proportions 0.07, 95% CI −0.04 to 0.17; p = 0.23).

### 3.4 IgA antibody titres to RV3-BB

Following three doses of RV3-BB vaccine co-administered with OPV, serum anti-rotavirus IgA titres were higher than in the placebo group, in both the neonatal (GMT 80.46 vs 25.32, geometric ratio 3.18, 95% CI 2.03–4.98, p < 0.001) and infant vaccine groups (GMT 139.3 vs 29.4, geometric ratio 4.74, 95% CI 3.07–7.31, p < 0.001) (Fig. 3C, D). Similarly, in those who received IPV, serum anti-rotavirus IgA titres were higher in the neonatal (GMT 98.94 vs 35.64, geometric ratio 2.78, 95% CI 1.51–5.09, p = 0.001) and infant (GMT 167.8 vs 45.7, geometric ratio 3.68, 95% CI 2.13–6.34, p < 0.001) compared with the placebo group.

There was little evidence of a difference in anti-rotavirus IgA titres in participants administered IPV compared to OPV following three doses of RV3-BB in either the neonatal (GMT 98.9 vs 80.5, geometric ratio 1.23, 95% CI 0.71–2.14, p = 0.463) or infant vaccine groups (GMT 167.8 vs 139.3, geometric ratio 1.20, 95% CI 0.74–1.96, p = 0.448) (Fig. 3C, D). After a single dose of RV3-BB in the neonatal group, anti-rotavirus IgA tires were similar in participants administered IPV group compared to OPV (GMT 21.1 vs 15.7, geometric ratio 1.34, 95% CI 0.89–2.01, p = 0.155). There, was however, evidence of a difference in anti-rotavirus IgA titres between those receiving IPV and OPV following 1 dose of placebo (GMT 18.5 vs 12.6, geometric ratio 1.47, 95% CI 1.00–2.17, p = 0.049) and a trend toward difference following 4 doses of placebo (GMT 45.65 vs 29.41, geometric ratio 1.55, 95% CI 0.94–2.56, p = 0.085).

## 4 Discussion

We observed that poliovirus serum antibody responses and serum antibody titres to poliovirus 1, 2 and 3 were similar in participants who received OPV co-administered with RV3-BB in either a neonatal or an infant schedule and participants who received placebo. This is consistent with that reported for co-administration of OPV with Rotarix [22], [15], [16], RotaTeq [6] and Rotavac [5]. The sero-protective rates and GMT to each poliovirus strain were high in all 3 treatment groups, similar to those previously reported in Yogyakarta, Indonesia [18]. The administration of the RV3-BB human neonatal rotavirus vaccine in a birth dose strategy is novel and has been demonstrated to be efficacious in a developing country setting [2]. Importantly, the findings of the current study demonstrate that implementation of RV3-BB in a birth dose strategy co-administered with a birth dose of OPV will not impact the global polio eradication program.

The co-administration of OPV and rotavirus vaccines has been evaluated in multiple settings and has been associated with lower anti-rotavirus IgA GMT, reduced seroconversion and stool shedding of the vaccine strain when compared to staggered vaccine administration [22], [15], [12], [8]. In our study we observed a trend toward lower serum anti-rotavirus IgA GMT when RV3-BB was co-administered with OPV compared with IPV. However, the evidence for this difference was weak, and importantly a similar trend was identified in the anti-rotavirus IgA GMT in the placebo groups. This suggests that this relationship may be explained by different exposure to wild-type rotavirus strains in the IPV and OPV groups.

Serum immune responses to Rotarix and the previously licenced Rotashield (Wyeth Laboratories, PA, USA) vaccines are lower when given at a younger age [17], [15]. The age difference in immune responses to Rotarix may have been influenced by the co-administration of OPV [15], [12]. Using a birth dose vaccination schedule we were unable to demonstrate evidence of a difference in anti-rotavirus IgA serological response or GMT between participants who received OPV and IPV. The lack of understanding of serological correlates of protection for rotavirus, potential interference of maternal antibodies and the immaturity of the immune system presents a challenge to understand the influence of OPV with a birth dose of RV3-BB. These difficulties are also shared with other vaccines administered in the newborn period [14]. Furthermore, the difference in the timing of serum collection from participants co-administered OPV (~4 weeks of age) and IPV groups (~8 to 10 weeks of age) may have confounded this comparison.

In conclusion, the co-administration of OPV with RV3-BB rotavirus vaccine in a birth dose strategy did not reduce the immunogenicity of either vaccine. These findings support the use of a neonatal RV3-BB vaccine in settings where either OPV or IPV is used in the routine vaccination schedule.

## Declaration of Competing Interest

CD is a fulltime employee of ViiV Healthcare, all work was completed whilst he was an employee at MCRI. CDK and MCRI hold a patent for the RV3-BB vaccine. RMS and NSB are employees of BioFarma PT who provided funds to support this study and plan to manufacture the RV3-BB vaccine. All other authors declare that they have no known competing financial interests or personal relationships that could have appeared to influence the work reported in this paper.
